# Supplementary figures and images for: A Structural Atlas of the Developing Zebrafish Telencephalon Based on Spatially-Restricted Transgene Expression
Source: Front Neuroanat. 2022 Jun 1;16:840924. doi: 10.3389/fnana.2022.840924 (PMC9198225; doi:10.3389/fnana.2022.840924)

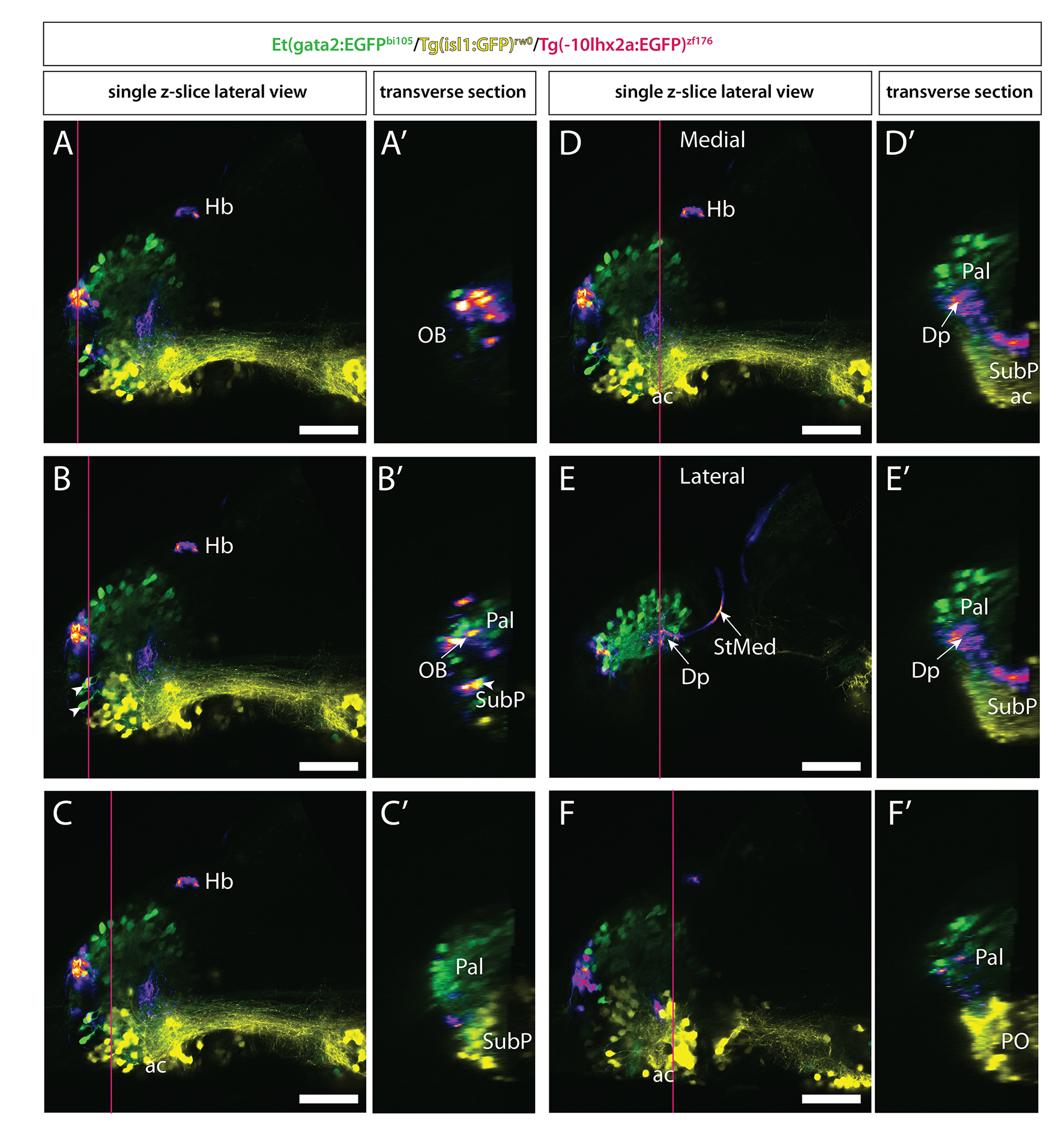

Supplement: Supplementary Figure 1 — Registered brain (lateral view) showing Et(gata2:EGFP)bi105 (green), Tg(-10lhx2a:EGFP)zf176 (FIRE) and Tg(isl1:GFP)rw0 (yellow) transgene expression at 5dpf. (A–F) Single z-slices showing transverse sections (A′–F′) made using orthogonal view tool (YZ) in ImageJ. Panel (E) is more lateral than panel (F). The rostro-caudal level of transverse sections (A′–F′) are indicated by a pink line on accompanying lateral z-slice in panels (A–F). (D,E) same rostro-caudal level, (D) closer z-slice to the midline than panel (E). (D′–E′) show the location of Tg(-10lhx2a:EGFP)zf176 mitral cell processes in putative Dp. Scale bars: 50 μm. [file Image_1.TIF]

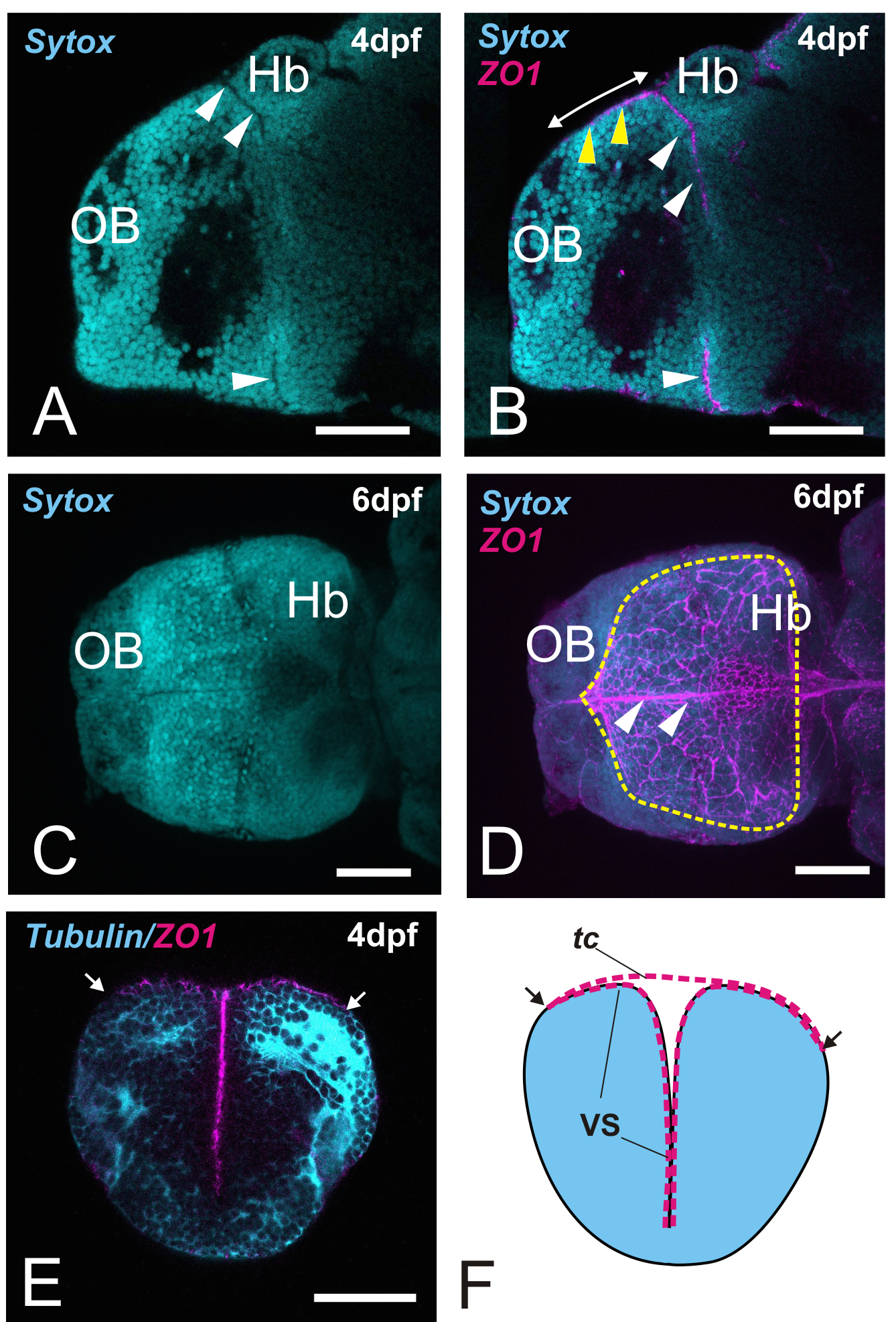

Supplement: Supplementary Figure 2 — ZO1 as a marker of the ventricular surface and tela choroidea. Lateral (A,B) and dorsal views (C,D) of the telencephalon of 4dpf (A,B) and 6dpf (C,D) fish stained against ZO1 (magenta) and counterstained with sytox orange (cyan). (A,B) White arrowheads point to the portions of the ventricle that are clearly visible with a nuclear stain only (AIS dorsally, optic recess ventrally). (B) Yellow arrowheads point to the extension of the ventricle visible by ZO1 staining. Double arrow marks the extension of the tela choroidea. This could be easily missed with a nuclear staining only [compare (A,B)]. (C,D) Full extension of the ventricle and tela choroidea is not clearly visible with a nuclear stain only in dorsal view (C), but clear when ZO1 staining is used (dotted line marks the full extension of the ventricle and tela choroidea dorsally). (E) Transverse section of the telencephalon of a 4dpf larva labeled against ZO1 (magenta) and tubulin (cyan). Note the T-shape of the ventricle. The tela choroidea extends dorsally (places of attachment or taeniae are marked with arrows). (F) Schematic representation of E, showing the ventricular surface (VS) and tela choroidea (tc) labeled by ZO1. Brain parenchyma is represented in pale blue. Taeniae marked with arrows. Scale bars: 100 μm. [file Image_2.TIF]

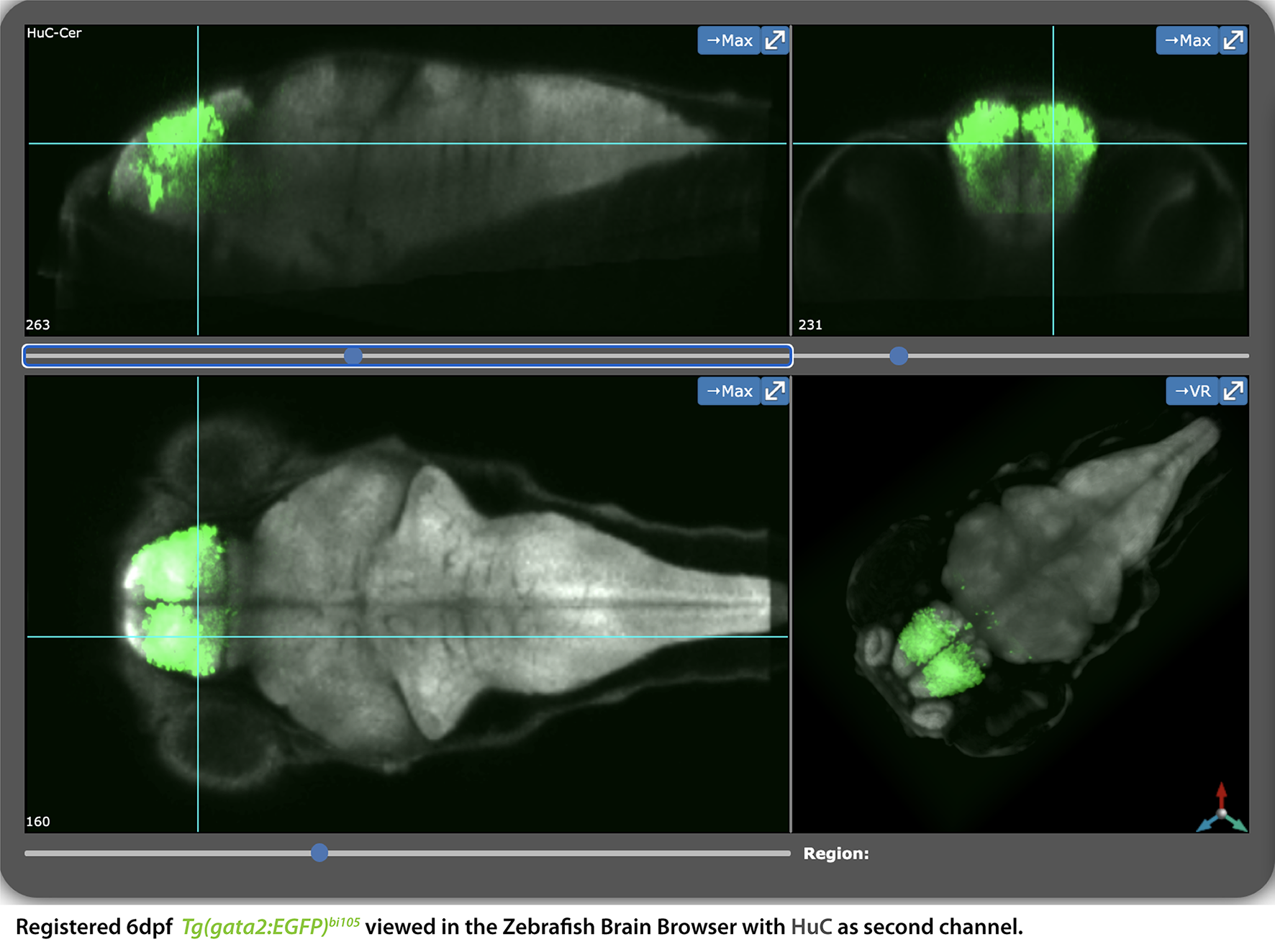

Supplement: Supplementary Figure 3 — Et(gata2:EGFP)bi105 expression at 6dpf registered to Zebrafish Brain Browser (ZBB). Screenshot of the online ZBB viewer showing a 6dpf Et(gata2:EGFP)bi105 larvae labeled with anti-EGFP (green) registered to the ZBB standard brain. HuC channel (gray) shows full brain structure. [file Image_3.TIF]

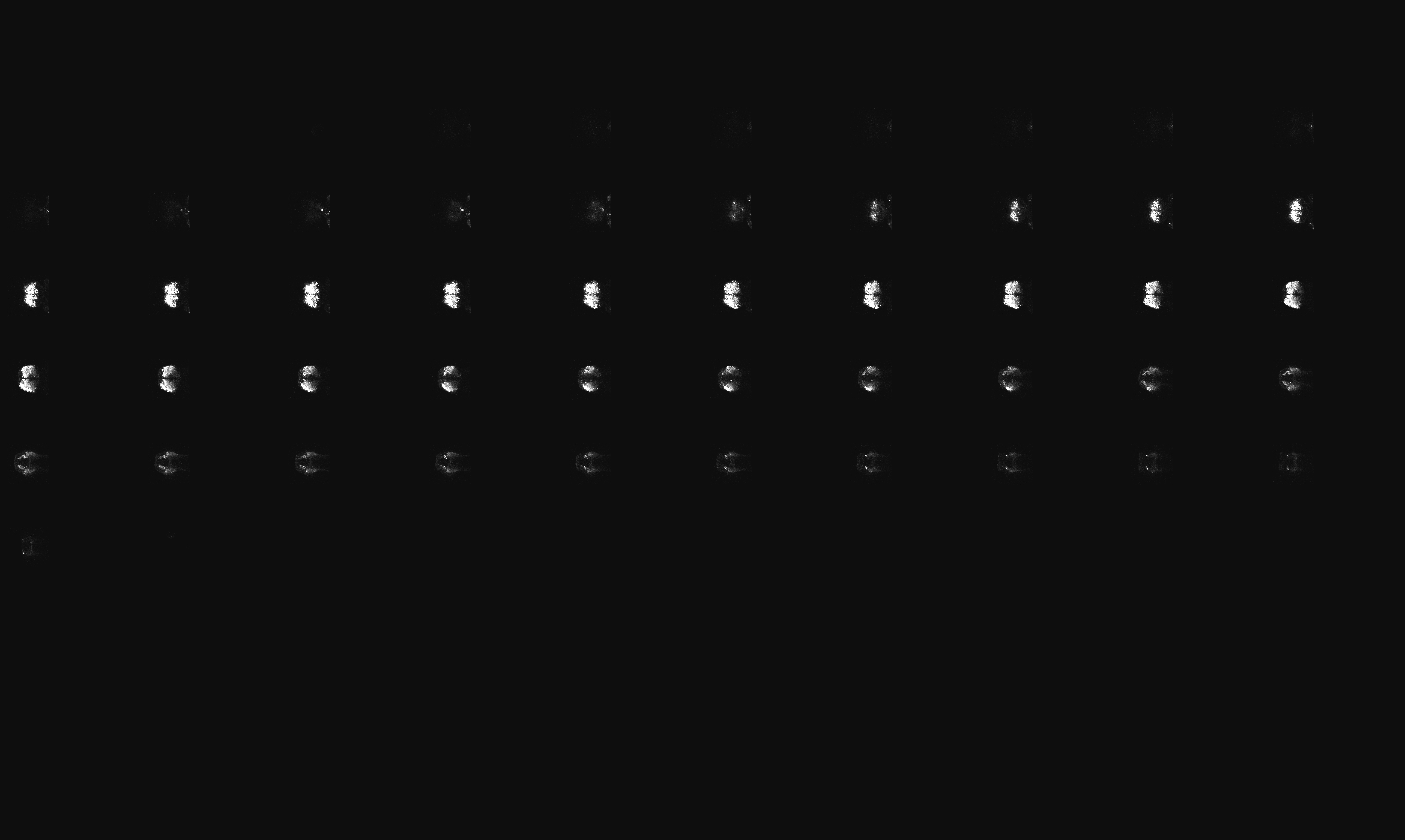

Supplement: Supplementary Figure 4 — A 6dpf Et(gata2:EGFP)bi105 stack in PNG format. This dataset can be uploaded to Zebrafish Brain Browser atlas (see “Materials and Methods”). [file Image_4.PNG]
